# Ultrastructure and Transcriptome Analysis of the Larval Integument in Solitary and Gregarious Phases of *Mythimna separata*

**DOI:** 10.3390/insects16020190

**Published:** 2025-02-10

**Authors:** Lingling Li, Wenmeng Li, Jing Liao, Junhong Fu, Changgeng Dai, Yang Hu, Hongbo Li

**Affiliations:** 1 Institute of Plant Protection, Guizhou Academy of Agricultural Sciences, Guiyang 550006, China; lilingling2019@163.com (L.L.); liaojing2025@126.com (J.L.); fujunhong231@sina.com (J.F.); ggyydai@163.com (C.D.); huyanggz@126.com (Y.H.); 2 Guizhou Branch of State Key Laboratory for Biology of Plant Diseases and Insect Pests, Guiyang 550006, China; 3 Key Laboratory of Forest Disaster Warning and Control in Yunnan Province, College of Forestry, Southwest Forestry University, Kunming 650224, China; lwmhbdgh1028@163.com; 4 Key Laboratory of Crop Genetic Resources and Germplasm Innovation in Karst Region, Ministry of Agriculture and Rural Affairs, Guiyang 550006, China

**Keywords:** *Mythimna separata*, integument ultrastructure, integument transcriptome, solitary phase, gregarious phase

## Abstract

*Mythimna separata* larvae exhibit both solitary and gregarious phases under low and high population density, respectively; furthermore, differences in morphology, behavior and physiology have been observed in the two phases. The integument plays an essential role in the fitness, general metabolism, communication, and survival of insects; however, differences in the integument ultrastructure and gene expression in the solitary and gregarious phases are largely unknown. In this study, the integument ultrastructure of larvae in the solitary and gregarious phases was compared, and transcriptome analysis was conducted to identify which genes were differentially expressed in the two phases. The results showed that the gregarious larvae had thicker integuments and more polygonal particles on the cuticle surface than solitary larvae. Using the Illumina HiSeq™ sequencing platform, 2774 differentially expressed genes (DEGs) were generated. Among these, many transcripts were identified with roles in the synthesis of fatty acids; structural components of the integument and the insecticide detoxification were differentially expressed in the integument of the two larval phases. qRT-PCR was used to validate expression patterns of the selected transcripts. This study provides a valuable resource for understanding the molecular basis of behavioral and physiological differences in the two phases of *M. separata*.

## Simple Summary

Phenotypic plasticity describes the ability of one genotype to produce different phenotypes in response to variable environments. The oriental armyworm, *Mythimna separata*, exhibits phenotypic plasticity in response to changes in population density. Solitary and gregarious phases occur at low and high larval densities, respectively, and lead to morphological, behavioral, and physiological differences. Gregarious larvae generally exhibit a darker body color, but differences in integument ultrastructure and gene expression in the two phases of *M. separata* are largely unknown. In this study, we compared both ultrastructure and differentially expressed genes in the integument of the two larval phases and verified the relevant results. We discovered that gregarious larvae had thicker integuments and more polygonal particles on the cuticle surface than solitary larvae. Many differentially expressed genes were identified relevant to fatty acid synthesis, integument structure, and insecticide detoxification. Our findings provide a valuable resource for understanding the mechanistic basis of behavioral and physiological differences in the two phases of *M. separata*.

## 1. Introduction

Phenotypic plasticity, which describes the ability of one genotype to produce different phenotypes in response to variable environments [1], is a widespread phenomenon in animals. When exposed to different environments, plastic traits will result in altered phenotypes in animals without genetic changes [2]. In general, phenotypes of animals are the outcome of interactions between genetic and environmental factors [3]. More importantly, altered phenotypes in animals are generally associated with higher fitness, which allows the animal to better survive in a rapidly changing environment [4,5,6]; consequently, phenotypic plasticity has become a hot topic in organismal ecology and evolution.

The oriental armyworm, *Mythimna separata*, is an important polyphagous insect pest that exhibits phenotypic plasticity in response to changes in population density. *M. separata* exhibits solitary and gregarious phases at low and high larval densities, respectively. In the past three decades, obvious differences in *M. separata* morphology, behavior, and physiology have been observed in the solitary and gregarious phases. Gregarious larvae generally exhibit smaller body sizes, a darker body color, and a stronger migratory capability when compared to solitary larvae [7,8]. At the physiological level, gregarious larvae have faster developmental rates, a stronger feeding capability, and a lower food utilization rate as compared to solitary larvae [7]. Most importantly, gregarious larvae display higher immunity and tolerance in response to fungal pathogen ingress [9,10,11], which may contribute to outbreaks in the field.

The insect integument consists of multiple layers that contain the cuticle and epidermal tissue. This protective outer layer covers the body surface and functions as a physical defense barrier against multiple stressors, including predators [12], pathogens [13], and other environmental stressors [14,15,16]. The cuticle contains an outer thin epicuticle and an inner thick procuticle. The epicuticle is composed of lipids and lipoproteins, while the procuticle is mostly formed by cuticular proteins and chitin. The epidermal tissue is a cell layer that underlies the insect cuticle and is primarily responsible for the formation of the epidermis and for external morphological changes that occur during insect growth and metamorphosis [12]. To adapt to specific environments, insects have evolved morphologically distinct structural and mechanical properties in their integument. For example, the melanic *Galleria mellonella* larvae have developed different melanin deposition patterns and thicker integuments when compared to non-melanic individuals, which enables them to better resist infection by the entomopathogenic fungus, *Metarhizium brunneum*, at the cuticle interface [13]. Furthermore, pesticide-tolerant insects were shown to develop thicker integument as compared to susceptible ones [14,15]. As described above, the gregarious larvae of *M. separata* exhibit different morphological, behavioral, and physiological traits as compared to solitary ones. However, differences in integument ultrastructure and the accompanying changes in gene expression are largely unknown for the solitary and gregarious phases of *M. separata* larvae.

In this study, the ultrastructure of the integument in solitary and gregarious *M. separata* was compared by scanning electron microscopy (SEM) and transmission electron microscopy (TEM). Furthermore, transcriptome sequencing was performed to identify genes that are differentially expressed in the integument of the two larval phases, and the results were verified by qRT-PCR. Our findings provide a valuable resource for understanding the mechanistic basis of behavioral and physiological differences in the two phases of *M. separata,* and will ultimately be helpful in understanding outbreaks in the field. 

## 2. Materials and Methods

### 2.1. Insects

The *M. separata* colony used in this study was originally collected in 2014 from corn fields located in Qianxi City, Guizhou, China. The solitary and gregarious phases of *M. separata* were established as described previously [11]. Briefly, solitary larvae were reared individually in a 300 mL cylindrical plastic container, and gregarious larvae were reared in a 1050 mL container at a density of 20 larvae per container. Both larval phases were maintained on a corn-based diet with a 14:10 h light/dark photoperiod at 25 ± 1 °C. After adults emerged, both phases were reared on a 10% honey/water diet.

### 2.2. Comparison of Larval Integument Properties in the Two Phases of M. separata

The appearance of fifth larval integuments in two phases of *M. separata* was initially photographed with a digital camera (DS-Fi3, Nikon, Tokyo, Japan). For SEM experiments, different integument regions (anterior, middle, and posterior) of the dorsal side were dissected from fifth instar larvae of solitary and gregarious phases. Dissected samples were washed with PBS, placed in fixative for 2 h at room temperature, and maintained at 4 °C until needed. The fixed samples were washed three times in 0.1 M PBS (pH 7.4) for 15 min. Samples were then incubated in 1% OsO_4_ in PBS (*v*/*v*) for 1–2 h at room temperature, followed by three more washes in PBS as described above. The fixed cuticles were dehydrated via immersion in a graded series of ethanol (30, 50, 70, 80, 90, and 95%, *v*/*v*) for 15 min each, transferred into 100% ethanol for 15 min (repeated twice), immersed in isoamyl acetate for 15 min, and placed in a critical point dryer. Samples were then attached to metallic stubs using carbon stickers and sputter-coated with gold for 30 s. Samples were then examined with by SEM (TM-1000, Hitachi, Tokyo, Japan). No obvious differences were observed in the ultrastructure of anterior, middle, and posterior regions of integument within the same phase; consequently, we decided to compare the middle regions of integument for differences in the two phases of *M. separata.* Five individuals from each phase were used for SEM analysis.

Integument thickness was compared in fifth instar larvae from the solitary and gregarious phases by TEM as described by Huang et al. [16]. Samples were observed by using a JEM-1210 transmission electron microscope (HT7800, Hitachi, Tokyo, Japan) at 80 kV. Our initial analysis did not reveal any remarkable differences in the cuticles of the two phases of *M. separata*; therefore, cuticular regions were randomly selected for further analysis. Digital images for each sample were recorded and used to measure thickness with Image J software (https://imagej.net/ij/ (accessed on 28 June 2012)). Five areas were selected in each larval integument, and each phase included four individuals.

### 2.3. RNA Extraction, Library Construction, and Illumina HiSeq™ Sequencing

Samples of both solitary and gregarious integument were collected from three fifth instar larvae. Each sample included three biological replications. Total RNA was extracted using the Eastep^®^ Super Total RNA Isolation Kit according to the manufacturer’s instructions (Promega, Madison, WI, USA), and genomic DNA was removed using DNase I (Promega, Madison, WI, USA). RNA quality and concentration were determined by electrophoresis in a 1.2% agarose gel with the Nanodrop Lite Spectrophotometer (Thermo Fisher Scientific, Waltham, MA, USA).

RNA purification, reverse transcription, library construction, and sequencing were conducted by the Bioyigene Sequencing Facility (Wuhan, China). The RNA-seq library was constructed using the NEBNext Ultra RNA Library Prep Kit as recommended by the manufacturer (New England Biolabs, Ipswich, MA, USA). Briefly, mRNA was isolated on oligo(dT) beads and fragmented, and cDNA was synthesized using the SuperScript Double-Stranded cDNA Synthesis Kit (Invitrogen, Carlsbad, CA, USA) with random hexamers (Illumina, San Diego, CA, USA). The synthesized cDNA was repaired, phosphorylated, and adenylated as recommended by Illumina. Libraries were size selected for 200–300 bp cDNA fragments in 2% low-range ultra-pure agarose and were PCR-amplified using Phusion DNA polymerase (New England Biolabs, Ipswich, MA, USA) for 15 cycles. PCR products were quantified with the TBS-380 fluorometer, and the paired-end RNA-seq library was sequenced using the Illumina HiSeq™ platform (HiSeq 2500). The raw paired-end reads were trimmed and the quality was analyzed using SeqPrep (https://github.com/jstjohn/SeqPrep (accessed on 5 October 2016)) and Sickle (https://github.com/najoshi/sickle (accessed on 4 August 2011)) with default parameters. Clean reads were separately aligned to the reference genome of *M. separata* [17] with TopHat v. 2.1.1 (http://tophat.cbcb.umd.edu/ (accessed on 1 April 2010)) [18].

### 2.4. Differential Expression Analysis and Functional Enrichment

To identify differentially expressed genes (DEGs) in the larval cuticles of the two phases, transcript expression levels were calculated according to the fragments per kilobase of exon per million mapped reads (FPKM). RNA-seq by Expectation-Maximization (RSEM; http://deweylab.biostat.wisc.edu/rsem/ (accessed on 14 February 2020)) was used to quantify gene abundance [19], and the R statistical package EdgeR (http://www.bioconductor.org/packages/2.12/bioc/html/edgeR.html (accessed on 1 January 2010)) was used for differential expression analysis [20]. GO (Gene Ontology) and KEGG (Kyoto Encyclopedia of Genes and Genomes) analyses were performed to identify DEGs significantly enriched in GO terms and metabolic pathways at *p* ≤ 0.05. GO functional enrichment and KEGG pathway analysis were conducted using ClusterProfiler (https://github.com/GuangchuangYu/clusterProfiler (accessed on 5 May 2012)) [21].

### 2.5. Quantitative Real-Time PCR

Quantitative real-time PCR (qRT-PCR) was conducted to verify the reliability of RNA-seq data; this analysis included genes involved in lipid biosynthesis (*n* = 16), genes encoding cuticular proteins (*n* = 10), and genes associated with pesticide resistance (*n* = 9) (Table S1). The first-strand cDNA was synthesized from total RNA of each sample using the protocols included with the iScript cDNA Synthesis Kit (Bio-Rad, Hercules, CA, USA). qRT-PCR was performed with the SsoAdvanced Universal SYBR Green Supermix (Bio-Rad, Hercules, CA, USA) and the CFX96 Real Time PCR System (Bio-Rad, Hercules, CA, USA) using the following parameters: 95 °C for 2 min, followed by 35 cycles of 95 °C for 5 s, and 30 s at 60 °C. Melting curve analysis was conducted to confirm primer specificity, *β-actin* was used as reference gene [22], and expression levels were calculated using the 2^−ΔΔCt^ method [23]. Each sample included three biological replications, and each replication had three technical replications.

### 2.6. Statistics

Statistical analyses were carried out using the DPS 17.0 statistical package [24]. Datapoints are shown as means ± standard error (SE). Differences in the solitary and gregarious phases were determined using a two-sample *t*-test with significance levels of *p* < 0.05 (*), *p* ≤ 0.01 (**), and *p* ≤ 0.001 (***).

## 3. Results and Discussion

### 3.1. Comparison of Larval Integument Properties in the Two Phases of M. separata

**Figure 1:**
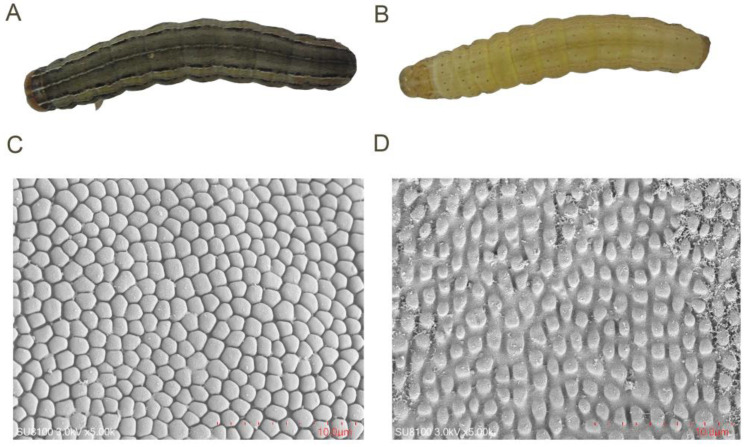
Integument properties in solitary and gregarious *M. separata* larvae. Integument appearance in gregarious (**A**) and solitary (**B**) larvae. Scanning electron microscopy showing differences in the morphology of polygonal particles in gregarious (**C**) and solitary (**D**) larvae.

**Figure 2:**
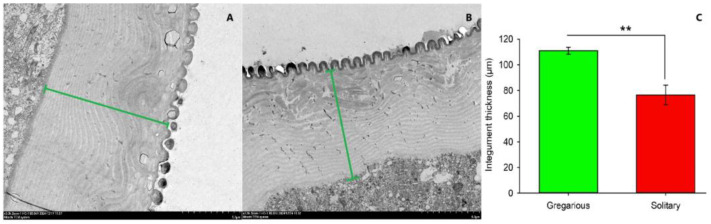
Comparison of integument thickness in different larval phases in *M. separata*. (**A**,**B**) Longitudinal section of the integument in gregarious and solitary larvae, respectively. Images were obtained by scanning electron microscopy. The green line indicates the integument thickness. (**C**) Mean integument thickness in the two phases of larvae using Image J software. Datapoints represent means (*n* = 4) ± standard error (SE). Asterisks indicate significant differences between solitary and gregarious larvae at *p* < 0.01 (**).

The integument of gregarious larvae was visibly darker in color than the integument of solitary individuals (Figure 1A,B). SEM analysis showed that more polygonal particles existed in the integument of gregarious larvae than solitary ones, and the edges of polygonal particles in the former was considerably sharper than that in the latter phase. Moreover, the distance between polygonal particles in gregarious individuals was smaller than that in solitary ones (Figure 1C,D). TEM analysis showed that the integument thickness of gregarious larvae (110.13 ± 2.59 µm) was thicker than that of solitary individuals (76.52 ± 7.75 µm) (Figure 2A–C). Similarly, Grizanova et. al. reported that the cuticle of melanic *G. mellonella* larvae was thicker and replete with more melanic spots than that of non-melanic larvae (NM) [13]. These features presumably reduced the ability of conidia to adhere to and germinate on the cuticular surface, which led to lower mortality in melanized larvae when compared to NM individuals [13]. In a previous study, gregarious *M. separata* larvae had higher survival rates than solitary ones when exposed to fungicides [9]. Therefore, we deduced that thicker cuticles in larvae with more particles on the surface contributed to the improved resistance of *M. separata* larvae to pathogens, thereby resulting in the relatively low effectiveness of fungal biopesticides in the field.

### 3.2. An Overview of the Integument Transcriptome in the Two Phases of M. separata

A total of 47.20 Gb of clean data were acquired using the Illumina HiSeq™ platform. The GC content ranged from 44.82% to 47.25%, and the Q30 rate ranged from 94.64% to 95.40% (Table S2). When these clean data were aligned to the reference genome of *M. separata* [17], a relatively high total mapped ratio (82.09% to 84.72%), multiple mapped ratio (3.61% to 7.20%), and uniquely mapped ratio (75.38% to 79.35%) were observed (Table S3). These results suggest that the quality of the data was high and could be used for further analysis.

### 3.3. Differential Expression Analysis and Functional Enrichment

**Figure 3:**
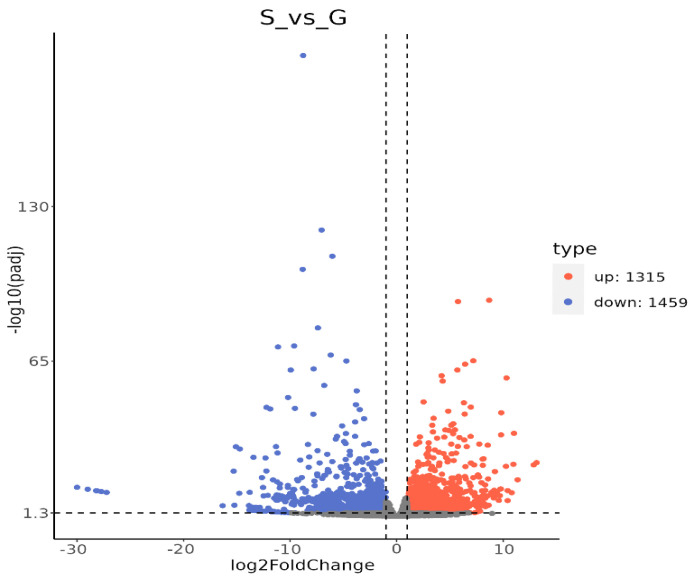
DEGs in the integument of solitary and gregarious *M. separata* larvae. The *x*-axis indicates log_2_-fold change and the *y*-axis indicates −log_10_ significance. Red and green dots represent up- and downregulated unigenes, respectively.

Comparative transcriptome analysis revealed that 2774 DEGs were identified in the integument of solitary and gregarious larvae. Among these, 1315 and 1459 genes were upregulated and downregulated in gregarious individuals, respectively (Figure 3).

**Figure 4:**
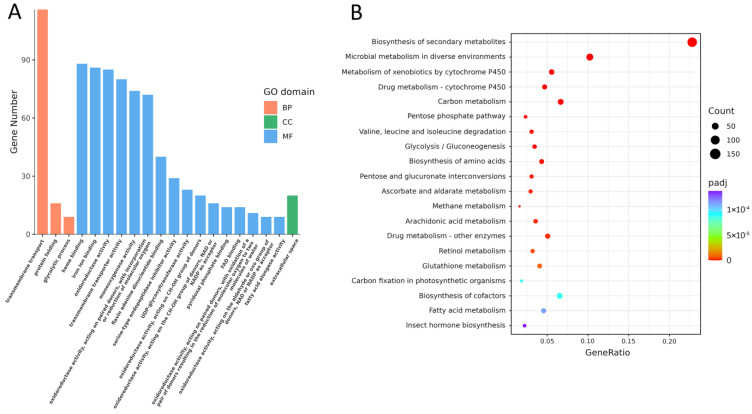
GO functional enrichment and KEGG analysis of DEGs. (**A**) GO analysis of the top 20 enriched pathways in the *M. separata* integument. The *x*-axis indicates the pathways, and the *y*-axis represents the number of DEGs represented in a particular pathway. Abbreviations: BP, biological processes; CC, cellular components; and MF, molecular functions. (**B**) KEGG analysis of the top 20 enriched pathways. The *x*-axis indicates the gene ratio, and the *y*-axis shows the categories. The size of the datapoints reflect a greater level of enrichment. The *q*-values are corrected *p*-values ranging from 0 to 1, with lower values indicating greater enrichment.

GO functional enrichment analysis of the DEGs was conducted with GOATOOLS (https://github.com/tanghaibao/goatools (accessed on 18 July 2018)), and the 2774 DEGs were classified into three categories, including “biological processes”, “molecular functions”, and “cellular components”. In the biological processes group, DEGs were enriched in “transmembrane transport”, “protein folding”, and “glycolytic process”. In the cellular components group, DEGs were enriched in the “extracellular space” group. With respect to molecular function, DEGs were enriched in “heme binding” and “iron ion binding”, followed by “oxidoreductase activity” and “transmembrane transport activity” (Figure 4A).

KEGG analysis showed that the 2774 DEGs could be subdivided into 251 pathways. Among these, gene expression levels in 51 different pathways were significantly changed in the integument of gregarious individuals as compared to solitary ones. These pathways included “biosynthesis of secondary metabolites”, “microbial metabolism in diverse environments”, “metabolism of xenobiotics by cytochrome P450”, “drug metabolism-cytochrome P450”, and “carbon metabolism” (Figure 4B). A description of the DEGs and their putative functions in *M. separata* is presented below.

#### 3.3.1. Genes Involved in Lipid Biosynthesis and Metabolism

**Figure 5:**
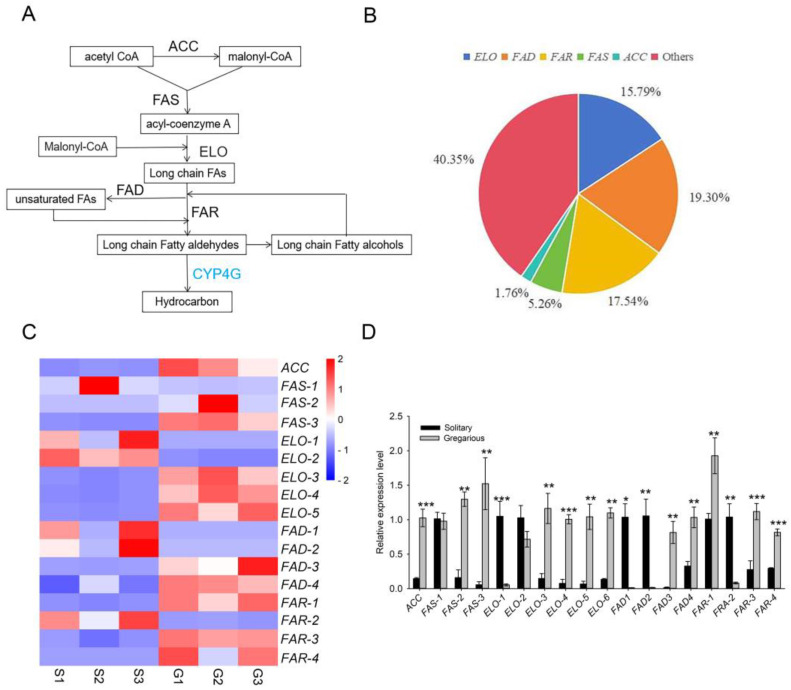
Genes involved in lipid biosynthesis and their expression in the larval integument of solitary and gregarious *M. separata*. (**A**) Diagram of the lipid biosynthesis pathway. Abbreviations: ACC, acetyl-CoA carboxylase; FAS, fatty acid synthase; ELO, elongase; FAD, fatty acid desaturase; and FAR, fatty acyl-CoA reductase. (**B**) Classification of 34 differentially regulated *M. separata* genes with roles in lipid biosynthesis. The pie chart shows relative percentages of the total, and frequencies included *ACC* (1.75%), *FAS* (5.26%), *ELO* (15.79%), *FAR* (17.54%), and *FAD* (19.39%). The other category had a frequency of 40.35% and included genes encoding aldehyde dehydrogenase, 3-hydroxyacyl-CoA dehydrogenase, short-chain 2-methylacyl-CoA dehydrogenase, and alcohol dehydrogenase. RNA-seq (**C**) and qRT-PCR (**D**) analysis of *ACC*, *FAS1*–*3*, *ELO1*–*5*, *FAD1*–*3*, and *FAR1*–*4* expression in the larval integument of solitary and gregarious *M. separata.* Expression levels in RNA-seq experiments were calculated according to the fragments per kilobase of exon per million mapped reads (FPKM) method. qRT-PCR results were normalized to *β-actin* using the 2^−ΔΔCt^ method. Datapoints represent means (*n* = 3) ± standard error. Asterisks indicate significant differences between solitary and gregarious larvae at *p* < 0.05 (*); *p* < 0.01 (**); and *p* < 0.001 (***).

The lipids present in the integument are a complicated mixture of nonpolar chemicals and consist of over 100 compounds, including hydrocarbons (HC), wax esters, fatty alcohols, and free or esterified fatty acids [25]. Among these, the HCs are the most abundant compound in insect lipids. The lipid composition in the integument is variable, and significant differences in composition have been observed among species, different development stages, and between sexes of the same species [26]. Lipid biosynthesis is highly conserved and can be classified into four distinct steps including the synthesis of fatty acid precursors, extension of fatty acid carbon chains, synthesis of very-long-chain fatty aldehydes, and dehydroxylation of very-long-chain fatty aldehydes and synthesis of hydrocarbons [27,28]. Enzymes involved in lipid synthesis include acetyl-CoA carboxylase (ACC), fatty acid synthase (FAS), elongase (ELO), fatty acid desaturase (FAD), fatty acyl-CoA reductase (FAR), and CYP4G (a cytochrome P450 subfamily), which finishes the synthesis of the hydrocarbon chain [25] (Figure 5A).

In this study, 57 transcripts were identified with putative roles in integument lipid metabolism, and 34 genes were associated with lipid biosynthesis pathways. The 34 genes linked to lipid biosynthesis and their relative percentages of the total included 1 gene encoding ACC (1.75%), 3 encoding FAS (5.26%), 9 encoding ELO (15.79%), 10 encoding FAR (17.54%), and 11 encoding FAD (19.39%) (Figure 4B, Table S4). Other genes with potential roles in lipid metabolism were 40.35% of the total number and included genes encoding aldehyde dehydrogenase, 3-hydroxyacyl-CoA dehydrogenase, short-chain 2-methylacyl-CoA dehydrogenase, and alcohol dehydrogenase (AD2) (Figure 5B).

#### 3.3.2. Acetyl-CoA Carboxylase

In the de novo hydrocarbon pathway, *ACC* converts acetyl-CoA to malonyl-CoA (Figure 4A), which functions as a substrate for fatty acid synthase [2]. Several studies have shown that *ACC* impacts lipid accumulation, reproduction, and cuticular functions in insects. For example, in *Aedes aegypti*, *ACC*-deficient mosquitoes had lower lipid content and produced defective oocytes that lacked intact eggshells due to *ACC* deficiencies in follicular epithelial cells [29]. In another study, the knockdown of *ACC* transcripts in fat bodies by RNA interference caused a dramatic reduction in triglyceride storage and a concurrent increase in glycogen accumulation in *Drosophila melanogaster* [30]. Furthermore, the mutation of *ACC*-encoding transcripts in oenocytes resulted in oenocyte mortality and caused a failure in the watertightness of spiracles, thus leading to the death of flies [30]. In *Nilaparvata lugens,* Zhang et al. [31] reported that the fungicide jinggangmycin improved reproduction and upregulated *ACC* transcription, whereas silencing *ACC* eliminated jinggangmycin-enhanced fecundity.

RNA-seq and qRT-PCR analysis confirmed that *ACC* was upregulated in gregarious larvae as compared to solitary individuals (Figure 5C,D). Previous studies showed that the upregulation of *ACC* was associated with insect resistance to selected pesticides [32,33]. Whether the upregulation of *ACC* contributes to enhanced pesticide resistance in gregarious larvae is unknown and warrants further study.

#### 3.3.3. Fatty Acid Synthases

Our results indicated that *FAS*-2 and *FAS*-3 were more highly expressed in the integument of gregarious *M. separata* larvae as compared to solitary individuals (Figure 5C,D). Biochemical experiments have demonstrated that insects contain both microsomal and cytosolic *FAS* [34,35]. The former enzyme is involved in the conversion of methyl-branched precursors to methyl-branched HCs, while the latter is involved in the synthesis and extension of cuticular hydrocarbons (CHCs) [35]. Two or three *FASs* have been reported in Drosophila [36] and related species including *Rhodnius prolixus* [37], *Triatoma infestans* [38], and *Locusta migratoria* [39]. Prior studies showed that Drosophila *FAS*^CG3523^ was primarily expressed in fat bodies, whereas *FAS*^CG3524^ and *FAS*^CG17374^ were predominantly expressed in the integument of oenocytes, suggesting that different *FAS* family members have divergent functions.

*FAS* function has been studied in several insect species. The RNAi-mediated knockdown of two *L. migratoria FAS* genes led to decreased hydrocarbon levels, reduced desiccation resistance, enhanced cuticular permeability, and increased sensitivity to insecticides [39]. In *R. prolixus* and *Blattella germanica* (L.), *FAS* knockdown reduced fatty acid and hydrocarbon levels in the integument, accelerated water loss, and led to the early death of individuals when subjected to desiccation [40,41]. Cheng et al. [42] showed that *FAS* suppression shortened the lifespan of *Laodelphax striatellus* adult females, inhibited the maturation of oocytes, reduced the expression of genes encoding vitellogenin and vitellogenin receptors, and decreased the production of offspring. In addition, *FAS* participated in cabbage beetle diapause by regulating the lipid accumulation and the expression of stress tolerance genes [43].

In some insects, the composition and amount of fatty acids in the integument contributes to differences in disease resistance [44,45,46,47]. We recently demonstrated that gregarious *M. separata* larvae exhibit stronger resistance to entomopathogenic fungi when compared to solitary larvae [9]. In this study, we found that two of the three *M. separata FAS* genes were highly expressed in the integument of gregarious larvae as compared to solitary individuals (Figure 5C,D). It is tempting to speculate that the upregulation of *FASs* contributes to enhanced disease resistance in gregarious larvae, but further evidence is needed to validate this hypothesis.

#### 3.3.4. Fatty Acyl-CoA Elongases

*ELO* expression in the integument of solitary and gregarious insects was compared by RNA-seq and qRT-PCR. There was variability among the different *ELO* alleles; for example, *ELO1* was upregulated in solitary larvae and downregulated in gregarious larvae. In contrast, there were no significant differences in the expression of *ELO2* in the two phases, whereas *ELO3*, *ELO4*, and *ELO-5* were significantly upregulated in gregarious larvae as compared to solitary larvae (Figure 5C,D).

Fatty acyl-CoA elongases are widely distributed in animals, plants, and microorganisms. In insects, *ELOs* play crucial roles in regulating the biosynthesis of hydrocarbons and sex pheromones and affect mating, fecundity, and cuticular functions [48,49,50,51]. *ELOs* have been identified in *D. melanogaster*, *Bombyx mori*, *Tribolium castaneum*, *Danaus plexippus*, *Anopheles gambiae*, *Apis mellifera*, *L. migratoria*, *Cyrtotrachelus buqueti*, and *N. lugens* [11,50,52,53]. The number of *ELOs* is variable among species, ranging from seven in *L. migratoria* [52] to 20 in *N. lugens* [54]. *ELO* proteins are characterized by a conserved domain composition, a transmembrane region, and highly conserved HxxHH and YxYY motifs [55].

#### 3.3.5. Fatty Acyl-CoA Reductases

Our RNA-seq and qRT-PCR data consistently indicated that *FAR2* was upregulated in the integument of solitary larvae and significantly downregulated in gregarious larvae (Figure 4C,D). Conversely, *FAR1*, *FAR3,* and *FAR4* were upregulated in gregarious larvae as compared to solitary larvae (Figure 5C,D). The precise roles of the *FAR* orthologs in the two phases are unknown and require further study.

In insects, *FAR* plays an important role in converting carboxylic acids to alcohol during pheromone biosynthesis. The role of *FARs* has been investigated in sex pheromone biosynthesis due to their high specificity and substrate selectivity [56,57,58]. In *B. mori*, *BmFAR* was specifically expressed in the pheromone gland, and functional experiments showed that *BmFAR* could catalyze the conversion of a sex pheromone precursor to form bombykol [59]. In the Adzuki bean borer, *Ostrinia scapulalis*, a pheromone-specific *FAR,* was involved in converting (Z)-11-tetradecenoic acid to the corresponding alcohol, (Z)-11-tetradecenol [60]. Recently, Cha and Lee found that the knockdown of a pheromone-gland-specific *FAR* reduced the production of a major sex pheromone component and decreased mating in *Maruca vitrata* moths [61].

#### 3.3.6. Fatty Acid Desaturases

*FAD1*, *FAD2*, *FAD3,* and *FAD4* exhibited contrasting expression patterns. For example, *FAD1* and *FAD2* were highly expressed in the integument of solitary *M. separata* larvae (Figure 5C,D). Interestingly, *FAD3* and *FAD4* were more highly expressed in gregarious larvae. The specific roles of the different *FAD* orthologs in *M. separata* is unknown and warrants further investigation.

*FADs* catalyze the formation of double bonds in the acyl chains of long-chain fatty acids. One O_2_ molecule and two electrons are required for the formation of each double bond, and the number of C atoms in the fatty acyl carbon chain and the position of the desaturated double bond are strictly selected [62,63].

The first insect *FAD* (*desat1*) was identified in *D. melanogaster* [64], and subsequent studies revealed a second allele, *desat2* [65]. Further analysis demonstrated that Desat1 showed specificity to palmitate, whereas Desat2 had substrate specificity for cardamom acid [65]. With the advent of genomic sequencing, large numbers of *FAD*s have been identified and characterized [66,67,68,69,70,71]. In addition to the fat body and oenocytes, *desat1* was abundant in testes, accessory glands, and ovaries, suggesting that this gene functions in both chemical communication via pheromones and also has a role in reproduction [72]. In soldier beetles, a *FAD* catalyzed the conversion of oleic acid to the 18 carbon precursor of 8 Z-dihydromatricaria acid, a chemical that beetles use to repel avian predators and protect eggs [73]. Collectively, these findings reveal the functional diversity of *FADs* in insects.

#### 3.3.7. Cuticular Proteins

Based on their chitin-binding domains (ChtBD) and conserved motifs, genes encoding cuticular proteins (*CP*) are classified into at least 13 families, including *CPR* (*CP* with Rebers and Riddiford (R&R) Consensus), CPAP, TWDL, CPF, CPF-like, CPT, CPLCA, CPLCG, CPLCW, CPLCP, CPCFC, CPG, and apidermin [74]. Many *CPs* have been characterized at the genomic, transcriptomic, or proteomic levels in different insect species and orders [75,76,77,78,79]. The number of characterized *CPs* varies among examined species, ranging from 63 *CPs* in *Pediculus humanuscorporis* to 305 *CPs* in *A. aegypti* [80]; this large range of *CP* genes may be due to differences in insect body sizes and habitats.

**Figure 6:**
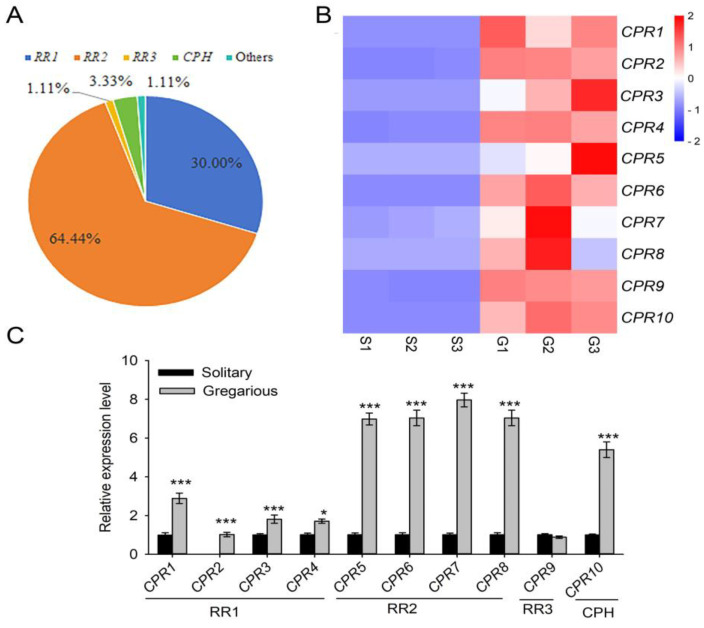
Genes encoding structural cuticular proteins (CPs) and their transcription in the larval integument of solitary and gregarious *M. separata.* (**A**) The classification of the 90 CPs in *M. separata* was based on the presence of the chitin-binding domain (ChtBD) and other conserved motifs. Out of 90 genes, 86 were further categorized into the following *CPRs* (CPs with the Rebers and Riddiford consensus) and included *RR-1* (*n* = 27), *RR-2* (*n* = 58), and *RR-3* (*n* = 1). The remaining *CPs* were categorized as *CPH* (*n* = 3) and ungrouped *CPs* (*n* = 1). The diagram shows relative percentages of the total, and frequencies included *RR-1* (30%), *RR-2* (64.4%), *RR-3* (1.11%), *CPH* (3.33%), and ungrouped others (1.11%). RNA-seq (**B**) and qRT-PCR (**C**) analysis of 10 *CPR* genes in the larval integument of solitary and gregarious *M. separata*. The expression level in RNA-seq was calculated as fragments per kilobase of exon per million mapped reads (FPKM). qRT-PCR data points indicate means (*n* = 3) ± standard error (SE). Asterisks indicated significant differences at *p* < 0.05, (*) and *p* < 0.001 (***).

Our analysis of the RNA-seq data identified 90 putative *CP* genes in the integument of solitary and gregarious *M. separata*. Based on their deduced amino acid sequence, the 90 *CPs* could be assigned to one of three subfamilies, e.g., *CPRs*, CPH (CP hypothetical), and ungrouped *CPs*. *CPR* was the most abundant subfamily in the larval integument of *M. separata* with 86 genes annotated, and these *CPR*-encoding genes could be further classified into the following subgroups: RR-1 (*n* = 27), RR-2 (*n* = 58), and RR-3 (*n* = 1). In addition to the 86 *CPR* genes, three *CPs* were annotated as CPHs, a family containing presumptive *CPs*. Furthermore, a single *CP* lacking the motifs characteristic of *CPs* was also identified (Figure 6A, Table S5).

Based on RNA-seq, we randomly selected 10 genes encoding *CPRs* (*CPR1-10*) and compared their expression profiles in the integument of solitary and gregarious *M. separata* larvae. RNA-seq analysis revealed that all 10 *CPR*s were more highly expressed in the integument of gregarious larvae than in solitary larvae. To verify the reliability of RNA-seq data, the 10 *CPRs* were subjected to qRT-PCR analysis. Our results showed that the expression profiles of *CPR1*–*8* and *CPR10* were consistent with the RNA-seq results; however, the expression of *CPR9* was not significantly different in the two *M. separata* phases (Figure 6B,C).

In *M. separata*, density-dependent prophylaxis has been observed, and many studies have focused on the immunological and stress response factors responsible for this phenomenon [9,81,82]. Some studies have reported that insects impair the damage caused by pathogen ingress or exposure to chemical pesticides by changing the cuticular structure or thickness [13,14]. Indeed, we found that the exoskeleton of gregarious larvae exhibited different thickness and surface structures than solitary larvae (Figure 1 and Figure 2). Therefore, we hypothesize that larval crowding can cause changes in the cuticular structure of gregarious larvae, and these changes lead to the upregulation of *CPs*, which ultimately enhances resistance to microorganisms in gregarious *M. separata* larvae.

#### 3.3.8. Genes Involved in the Metabolism and Detoxification of Insecticides

Insecticides are widely used to control pests and improve crop yield; however, the extensive use of insecticides has resulted in the development of pesticide-resistant insect populations. The enhanced detoxification and metabolism of insecticides are often the mechanistic basis for resistance, and these processes are often mediated by enzymes such as cytochrome P450s, carboxylesterases, glutathione-S-transferases, and UDP-glucuronosyltransferases [83,84]. Insecticide resistance in field populations of *M. separata* have been observed in China [85,86], but the underlying mechanisms are not completely understood.

**Figure 7:**
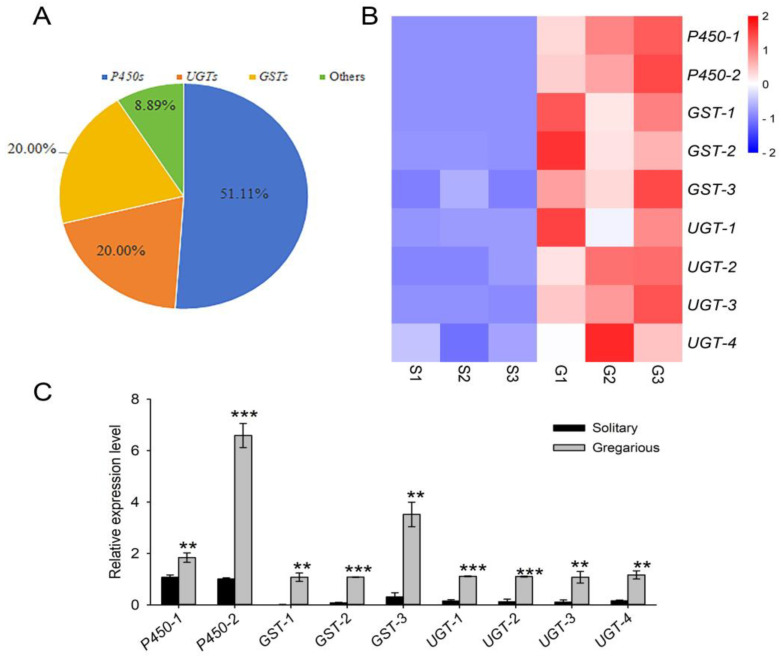
Genes involved in the detoxification and metabolism of insecticides and their expression in the integument of solitary and gregarious *M. separata* larvae. (**A**) Classification of nine genes associated with detoxification and metabolic insecticide resistance. RNA-seq (**B**) and qRT-PCR (**C**) analysis of *CYP4C1*–*2*, *GST1*–*3,* and *UGT1*–*4* in the larval integument of solitary and gregarious *M. separata*. Expression levels in the RNA-seq experiments were calculated according to the FPKM method described above. qRT-PCR results were normalized to *β-actin* expression levels and calculated according to the 2^−ΔΔCt^ method. Datapoints represent means (*n* = 3) ± standard error (SE). Asterisks indicated significant differences between solitary and gregarious larvae as follows: *p* < 0.01 (**) and *p* < 0.001 (***).

In this study, 88 transcripts with putative roles in insecticide resistance were differentially expressed in the solitary and gregarious phases of *M. separata*. Among these, genes encoding cytochrome P450 (*P450*, *n* = 46), UDP-glucuronosyltransferase (*UGT*, *n* = 17), and glutathione-S-transferase (*GST*, *n* = 17) accounted for 51.11%, 19.31, and 19.31% of the total, respectively; other genes accounted for the remaining 9.09% (Figure 7A, Table S6). RNA-seq and qRT-PCR analysis of the nine genes (e.g., *CYP4C1*–*2*, *GST1*–*3,* and *UGT1*–*4*) showed that all were significantly upregulated in the integument of gregarious larvae as compared to that of solitary larvae (Figure 7B,C). The insect integument acts an efficient barrier against adhered pesticide drops, and its role as a penetration factor has been previously reported in several species [15,87,88]. Interestingly, recent studies have demonstrated that several *P450*s were highly expressed in the integument of resistant *Triatoma infestans* nymphs, and the silencing of these genes by RNA interference caused a significant increase in mortality after exposure to deltamethrin [89,90]. Similarly, the knockdown of an integument-enriched esterase gene, *LbEST-inte4*, resulted in a substantial increase in susceptibility to malathion in *Liposcelis bostrychophila* [91]. These results suggest that the insect integument plays an important role in metabolic resistance. Therefore, we hypothesize that upregulation of *P450*s, *GST*s, and *UGT*s might contribute to resistance in the integument tissue of gregarious larvae in *M. separata*. Further functional analysis such as suppression by RNAi is needed to test this hypothesis.

## 4. Conclusions

In summary, SEM and TEM analysis showed key differences regarding the morphology of the integument in gregarious and solitary *M. separata*. First, the gregarious larval phase exhibited a significantly thicker cuticle than solitary forms. Second, the gregarious phase had a higher density of polygonal particles on the integument surface when compared to solitary larvae. Furthermore, the polygonal particles in gregarious larvae had sharper edges and larger interparticle spaces than solitary larvae. Transcriptome analysis showed that a large number of genes involved in fatty acid synthesis (*ACC*, *FAS*, *ELO*, *FAR,* and *FADs*), integument structure (primarily *CPs*), and insecticide detoxification (*P450s*, *GSTs,* and *UGTs*) were differentially expressed in the integument of solitary and gregarious *M. separata*. These results provide a foundation for future studies focusing on the underlying behavioral and physiological differences in the two phases of *M. separata* larvae.

## Data Availability

Data are contained within the article.

## Supplementary Materials

The following supporting information can be downloaded at https://www.mdpi.com/article/10.3390/insects16020190/s1, Table S1: List of primers used in qRT-PCR experiments. Table S2: Sequencing data obtained from transcriptome analysis of integument in gregarious (G) and solitary (S) *M. separata*. Table S3: Comparison of sequencing data from transcriptome analysis of gregarious (G) and solitary (S) *M. separata* vs. the *M. separata* reference genome. Table S4: List of differentially expressed genes involved in lipid biosynthesis in the larval integument of solitary and gregarious *M. separata*. Table S5: List of differentially expressed cuticular proteins in the larval integument of solitary and gregarious *M. separata*. Table S6: List of differentially expressed genes associated with pesticide resistance in the larval integument of solitary and gregarious *M. separata*. Sequenced raw reads were deposited in GenBank as accession number PRJNA1171735.
