# Supplementary material for: Ultrastructure and Transcriptome Analysis of the Larval Integument in Solitary and Gregarious Phases of Mythimna separata
Source: Insects. 2025 Feb 10;16(2):190. doi: 10.3390/insects16020190 (PMC11856551; doi:10.3390/insects16020190)
Supplement: Supplementary file 1 [file insects-16-00190-s001.zip › insects-3327744-supplementary.pdf]

Table S1. List of primers used in qRT-PCR experiments.

Primer name	Forward sequence (5'-3')	Reverse sequence (5'-3')
ACC	GCCCGTAACGACTTCACAGA	CTTCACCTTAGCCTTGCCCA
FAR-1	GATTTGATGAGCCGCTTCG	GCGTGTGAGTATGCCGTTGA
FAR-2	TGAAGACCTGGTTGCGGAGTA	GTCCGTTGAGATTGTCCACCC
FAR-3	CAGCCACCGTGAACTTCCAA	GTGACACTGCTTGCCCAACT
FAR-4	GGACATTCTCGCTGACCAGGT	CGTGTTGATCATAGTCGCCGC
FAD-1	GACTACAAGGCAGCCGAACTC	AGAAGCCCATCCCATCCTCTC
FAD-2	GGCGAGGCAGTATTTCCATCC	GCAGCACTATTGACGAGCCAT
FAD-3	GCCGTTACCACCTCATCCAA	AGTGTTGTGGCTGTGTGGAA
FAD-4	GAAGCCGCCTACTCCTATGGA	CGCTTTCCATCCCCACAACTT
ELO-1	CCCAGGAGGTCACACAATCC	TCCACCACACGTACTTCTGG
ELO-2	TCCACGTCTACCACCACACTT	TCCCAGGAACAGCAGAGCATA
ELO-3	AAGCGAGACGGATGTTACACC	CTTCAAAAGGCGTCAGGGAGT
ELO-4	CCGACAATCCTAGAGCACTCAG	TGGTGTCCAGAAGTTCCGTGA
ELO-5	GCAGTATCTTGGGGCATCGTC	TGGTCAGCAAGTAGTAGCCGT
ELO-6	GCTGGTCTGGGTCCTCAATAC	CAATCACGACGAGAAACTGCG
FAS-1	CAAGACGTGGTTGCACTTCG	ACTGGACTTGTAGCGGTTGG
FAS-2	CGGTTCGATGCCCAGTTCTT	TGCTCAAGTCCCTTCCGTGA
FAS-3	GCCCAGAGATATGCAAAGGTCC	AGTGCCACGAATACTCCTGTCT
CYP4C1	CACCCGCTATTGTACCTGGAG	GCCGAACCAAGACTCACAAAC
CYP4C2	TGTTACTCGGACTTCACTGGGA	TAGCCACCAACTCATCGATTGC
GST-1	GGGAGGAGAACACTTAACGCC	TCAGCAAGGAGGAAGTCACCA
GST-2	CGAGACTGATGAAGCTGTGAAGG	GTTAGGACGAAATCTGCCCAGG
GST-3	CCTCGGAAAGTTGACATGGGG	ACTTCTTCTCAAGGTCGGGCA
GST-4	GACCAGTTTCTCAGCAACAGC	GAGCAACTCTTCACCCATCGT
UGT-1	TCAAGGAGGTCTGCAATCGAC	ACTGGTCTCCTAACATCGGCA
UGT-2	TCACCGTCGTCACAACAGATC	CGATGCTACAATGTCGTTCGC
UGT-3	GAAACTGGAGCTGGCATCACTT	ACGGTCTAGAGGCTTCATTGGT
UGT-4	ACCAATCCTGCTTACGACCCT	TTTGACCCTGGCCCACAATTC
CP1	CCGGTCCAAATTCGATTCGC	GGGGCTTGTTCTCCTCATCC
CP2	GGATGAGGAGAACAAGCCCC	AAGGTCCCTCGGCATGGTAA
CP3	CTGTCCTGGATTCTGAAGGCA	GACTTTCCTTCTGCGTGGTAG
CP4	CGTCCGGTCCAACTTCGATT	CTCTTCACTTCACCCGCCTC
CP5	CGGACTACACCCACTTTGCT	AACAGCGGCATCTTTACGGA
CP6	AGCGGCTATCCCTACCATCT	CTGCCTGTAGTTGGTCCTGG
CP7	AGCTCGTCAAGGTCCGTTACT	AGTTCAGGGGTCTCTTCTGCA
CP8	CTTCCTCCATTCCGTTCCAAC	TTAGCAGAAGGTCCGTGGTCA
CP9	AAGTTCGTCGCACTCTTCGC	ATCAGCCACGCCGTATGAGAA
CP10	TCCAACCAGTACCTGCCTCC	TGTTAGGGAGCCAGCGAACT
Actin	AACTTCCCGACGGTCAAGTCAT	TGTTGGCGTACAAGTCCTTACG

Abbreviations: ACC, acetyl-CoA carboxylase; FAR, fatty acyl-CoA reductase; FAD, *fatty acid desaturase; ELO, fatty acyl-CoA elongase*; FAS, fatty acid synthase; *CYP4C,* cytochrome P450 4C subfamily; GST, glutathione-S-transferases; UGT, UDP-glucuronosyltransferase; CP, cuticular protein.

Table S2. Sequencing data obtained from transcriptome analysis of integument in gregarious (G) and solitary (S) *M. separata*.

Sample	Total reads	Total bases	Clean reads	Clean bases	Q20 (%)	Q30 (%)	GC (%)
G1	49910710	7486606500	49910708	7352239236	99.00	95.26	45.29
G2	51323748	7698562200	51323746	7585043874	99.03	95.4	45.75
G3	53588638	8038295700	53588638	7939625396	98.97	95.24	45.59
S1	45758518	6863777700	45758516	6778791766	98.76	94.64	47.25
S2	37519706	5627955900	37519702	5543546672	98.9	94.93	44.82
S3	80919526	12137928900	80919524	11998392278	98.81	94.78	46.47

Table S3. Comparison of sequencing data from transcriptome analysis of gregarious (G) and solitary (S) *M. separata* vs. the *M. separata* reference genome.

Sample	Total reads	Total mapped	Multiple mapped	Uniquely mapped
G1	49910708	41549982 (83.25%)	2798702 (5.61%)	38751280 (77.64%)
G2	51323746	42857133 (83.50%)	3051681 (5.95%)	39805452 (77.56%)
G3	53588638	45399723 (84.72%)	2876607 (5.37%)	42523116 (79.35%)
S1	45758516	37766485 (82.53%)	2969982 (6.49%)	34796503 (76.04%)
S2	37519702	30801670 (82.09%)	1355888 (3.61%)	29445782 (78.48%)
S3	80919524	66826143 (82.58%)	5827607 (7.20%)	60998536 (75.38%)

Table S4. List of differentially expressed genes involved in lipid biosynthesis in the larval integument of solitary and gregarious *M. separata.*

Gene name	Gene ID	S-RPKM	G-RPKM	Log_2 ratio(G/S)	Annotation
ACC	PYW07_011453	606.10	3494.35	2.53	Acetyl-CoA carboxylase [*M. separata*]
FAS1	PYW07_008655	0.43	417.16	9.60	Fatty acid synthase [*Heliothis virescens*]
FAS2	PYW07_013610	1035.66	21793.74	4.40	Fatty acid synthase [*Agrotis ipsilon*]
FAS3	PYW07_008650	2923.97	523.98	-2.48	Fatty acid synthase-like isoform X1 [*Spodoptera litura*]
FAS-4	PYW07_008654	23.25	190.73	3.04	Fatty acid synthase [*Helicoverpa armigera*]

ELO-1	PYW07_015079	417.14	1.03	-8.65	Elongation of very-long-chain fatty acids protein[*Spodoptera litura*]
ELO-2	PYW07_015084	596.18	50.01	-3.58	Elongation of very-long-chain fatty acids protein [*Helicoverpa armigera*]
ELO-3	PYW07_015074	41.16	1480.75	5.17	Elongation of very-long-chain fatty acids protein AAEL008004-like [*Helicoverpa armigera*]
ELO-4	PYW07_007999	88.66	3201.02	5.17	Elongation of very-long-chain fatty acids protein [*Spodoptera litura*]
ELO-5	PYW07_015075	28.96	2754.80	6.57	Elongation of very-long-chain fatty acids protein [*Heliothis virescens*]
ELO-6	PYW07_007999	88.66	3201.02	5.17	Elongation of very-long-chain fatty acids protein 7-like [*Spodoptera litura*]
ELO-7	PYW07_008610	0.95	303.14	1.96	Elongation of very-long-chain fatty acids protein AAEL008004-like [*Spodoptera frugiperda*]
ELO-8	PYW07_015072	1116.35	173.10	-2.69	Elongation of very-long-chain fatty acids protein AAEL008004-like isoform X2 [*Helicoverpa armigera*]
ELO-9	PYW07_015427	3772.44	440.95	-3.10	Elongation of very-long-chain fatty acids protein AAEL008004 [*Trichoplusia ni*
FAR-1	PYW07_011426	1478.50	2.06	-9.48	Fatty acyl-CoA reductase 12, partial [*Helicoverpa assulta*]
FAR-2	PYW07_011262	3623.13	269.91	-3.75	Putative fatty acyl-CoA reductase CG5065 [*Helicoverpa armigera*]
FAR-3	PYW07_011258	512.48	108.03	-2.25	Fatty acyl-CoA reductase 1-like [*Spodoptera frugiperda*]
FAR-4	PYW07_011265	691.87	175.46	-1.98	Fatty acyl-CoA reductase wat-like [*Helicoverpa armigera*]
FAR-5	PYW07_012432	173.58	4694.48	4.76	Fatty acyl-CoA reductase wat-like [*Spodoptera frugiperda*]
FAR-6	PYW07_012432	173.58	4694.48	4.76	Fatty acyl-CoA reductase wat-like [*Spodoptera frugiperda*]
FAR-7	PYW07_011169	0.79	582.92	9.78	Fatty acyl-CoA reductase wat-like [*Spodoptera litura*]
FAR-8	PYW07_006377	10.18	583.88	5.82	Fatty acyl-CoA reductase wat-like [*Helicoverpa armigera*]
FAR-9	PYW07_006378	390.53	3305.24	3.08	Fatty acyl-CoA reductase wat-like [*Helicoverpa armigera*]
FAR-10	PYW07_011252	8.94	342.10	5.30	fatty acyl-CoA reductase wat-like [*Helicoverpa armigera*]
FAD-1	PYW07_016499	4216.83	0.00	-28.14	Acyl-CoA Delta(11) desaturase-like [*Spodoptera litura*]
FAD-2	PYW07_016500	3085.43	0.00	-27.71	Acyl-CoA Delta(11) desaturase-like [*Spodoptera litura*]
FAD-3	PYW07_002780	7765.59	0.00	-15.30	Acyl-CoA Delta-9 desaturase [*Acheta domesticus*]
FAD-4	PYW07_016501	1031.07	0.00	-12.39	Acyl-CoA Delta(11) desaturase-like [*Spodoptera litura*]
FAD-5	PYW07_016502	668.92	0.00	-11.76	Acyl-CoA Delta(11) desaturase-like [*Spodoptera litura*]
FAD-6	PYW07_016498	502.83	0.00	-11.35	Stearoyl-CoA desaturase (Delta-9 desaturase)[*Heliothis virescens*]
FAD-7	PYW07_002329	382.97	151.14	-1.34	Stearoyl-CoA desaturase (Delta-9 desaturase) [*Agrotis segetum*]
FAD-8	PYW07_011342	84.19	292.71	1.80	Putative desaturase des8 [*Spodoptera litura*]
FAD-9	PYW07_002235	1897.16	16118.66	3.09	Acyl-CoA delta 9 desaturase [*Mamestra brassicae*]
FAD-10	PYW07_002342	4391.71	46733.42	3.41	Desaturase [*Agrotis segetum*]
FAD-11	PYW07_002338	4.15	524.98	6.94	Acyl-CoA Delta(11) desaturase-like [*Spodoptera litura*]

Table S5. List of differentially expressed cuticular proteins in the larval integument of solitary and gregarious *M. separata*.

Gene name	Gene ID	S-RPKM	G-RPKM	Log2 ratio (G/S)	Annotation
CPR1-RR1	PYW07_015330	118.70	107533.97	9.82	Larval cuticle protein RR1 [*Helicoverpa armigera*]
CPR2-RR1	PYW07_015331	877.08	174270.26	7.63	Larval cuticle protein RR1 [*Spodoptera litura*]
CPR3-RR1	PYW07_015329	12.62	1925.27	7.27	Larval cuticle protein RR1 [*Helicoverpa armigera*]
CPR4-RR1	PYW07_015332	3665.95	539576.06	7.20	Larval cuticle protein RR1 [*Helicoverpa armigera*]
CPR5-RR1	PYW07_015323	597.15	5075.89	3.09	Larval cuticle protein LCP-17-like [*Spodoptera litura*]
CPR6-RR1	PYW07_005554	2174.10	0.00	-13.46	Pupal cuticle protein 36a-like [*Helicoverpa armigera*]
CPR7-RR1	PYW07_017023	282.42	0.00	-10.52	Pupal cuticle protein 20 [*Manduca sexta*]
CPR8-RR1	PYW07_005572	41145.31	35.13	-10.19	Cuticle protein 3-like [*Helicoverpa armiger*a]
CPR9-RR1	PYW07_005560	1827.56	2.55	-9.49	Pupal cuticle protein 36a-like [*Spodoptera litura*]
CPR10-RR1	PYW07_015318	341635.12	828.22	-8.69	Larval cuticle protein LCP-22-like [*Spodoptera frugiperda*]
CPR11-RR1	PYW07_015303	28.44	0.00	-7.20	Endocuticle structural glycoprotein SgAbd-5 [*Heliothis virescens*]
CPR12-RR1	PYW07_005556	957.28	8.99	-6.73	Pupal cuticle protein 36-like [*Helicoverpa armigera*]
CPR13-RR1	PYW07_005561	4014.64	37.73	-6.73	Pupal cuticle protein 36a-like [*Spodoptera litura*]
CPR14-RR1	PYW07_015310	186269.79	2251.57	-6.37	Endocuticle structural glycoprotein SgAbd-2 [*Schistocerca gregaria*]
CPR15-RR1	PYW07_015297	1636.53	20.01	-6.35	Endocuticle structural glycoprotein SgAbd-5-like [*Helicoverpa armigera*]
CPR16-RR1	PYW07_005553	2519.06	37.49	-6.07	Pupal cuticle protein 36 [*Manduca sexta*]
CPR17-RR1	PYW07_005558	1139.35	17.92	-5.99	Pupal cuticle protein 36-like [*Helicoverpa armigera*]
CPR18-RR1	PYW07_015324	13188.27	256.25	-5.69	Endocuticle structural glycoprotein SgAbd-8-like [*Helicoverpa armigera*]
CPR19-RR1	PYW07_005555	4801.75	104.57	-5.52	Pupal cuticle protein 36-like [*Helicoverpa armigera*]
CPR20-RR1	PYW07_015311	723.92	17.59	-5.36	Endocuticle structural glycoprotein ABD-4-like [*Spodoptera frugiperda*]
CPR21-RR1	PYW07_005573	31.34	1.37	-4.51	Cuticle protein 3 [*Lonomia obliqua*]
CPR22-RR1	PYW07_015325	2480.29	115.87	-4.42	Endocuticle structural glycoprotein SgAbd-8-like [*Spodoptera frugiperda*]
CPR23-RR1	PYW07_015312	29045.29	1924.18	-3.92	Endocuticle structural glycoprotein SgAbd-8 [*Schistocerca gregaria*]
CPR24-RR1	PYW07_005564	4112.90	299.08	-3.78	Pupal cuticle protein 36-like [*Spodoptera litura*]
CPR25-RR1	PYW07_005575	372.50	27.31	-3.77	Pupal cuticle protein 36a [*Manduca sexta*]
CPR26-RR1	PYW07_007052	8425.82	2357.48	-1.84	Larval cuticle protein LCP-30-like [*Helicoverpa armigera*]
CPR27-RR1	PYW07_001328	152.99	5405.66	5.14	Cuticle protein 16.5-like isoform X1 [*Helicoverpa armigera*]
CPR28-RR2	PYW07_005568	16.34	277.39	4.07	Pupal cuticle protein 36-like isoform X1 [*Helicoverpa armigera*]
CPR29-RR2	PYW07_005569	56.26	440.67	2.97	Pupal cuticle protein 36-like isoform X1 [*Helicoverpa armigera*]
CPR30-RR2	PYW07_008991	24230.38	0.00	-30.00	Larval cuticle protein A2B [*Tenebrio molitor*]
CPR31-RR2	PYW07_008999	7851.71	0.00	-29.00	Cuticle protein [*Anopheles gambiae*]
CPR32-RR2	PYW07_015472	4748.42	0.00	-28.20	Cuticle protein 8 [*Spodoptera litura*]
CPR33-RR2	PYW07_002891	4120.55	0.00	-28.12	Cuticle protein 18.6 [*Locusta migratoria*]
CPR34-RR2	PYW07_015475	3303.01	0.00	-27.23	Cuticle protein 8-like isoform X1 [*Helicoverpa armigera*]
CPR35-RR2	PYW07_008992	15892.51	0.00	-16.33	Larval cuticle protein A1A [Tenebrio molitor]
CPR36-RR2	PYW07_008993	10535.74	0.34	-14.78	Larval cuticle protein A2B-like [*Spodoptera litura*]
CPR37-RR2	PYW07_002890	2868.10	0.00	-13.86	Cuticle protein 8 [*Locusta migratoria*]
CPR38-RR2	PYW07_015471	2798.74	0.00	-13.83	Cuticle protein 8 [*Locusta migratoria*]
CPR39-RR2	PYW07_008998	2397.62	0.00	-13.60	Cuticle protein [*Anopheles gambiae*]

CPR40-RR2	PYW07_015462	2285.32	0.00	-13.53	Cuticle protein 8-like [*Spodoptera frugiperda*]
CPR41-RR2	PYW07_007978	2260.89	0.00	-13.52	Cuticle protein 8-like [*Helicoverpa armigera*]
CPR42-RR2	PYW07_015452	1908.01	0.00	-13.27	Cuticle protein 7-like [*Spodoptera frugiperda*]
CPR43-RR2	PYW07_015467	1837.28	0.00	-13.22	Cuticle protein 8-like [*Spodoptera frugiperda*]
CPR44-RR2	PYW07_015453	1764.93	0.00	-13.16	Cuticle protein 8-like [*Helicoverpa armigera*]
CPR45-RR2	PYW07_015468	1744.16	0.00	-13.14	Cuticle protein-like [*Spodoptera frugiperda*]
CPR46-RR2	PYW07_008997	5947.42	0.68	-13.08	Larval cuticle protein A2B-like [*Helicoverpa armigera*]
CPR47-RR2	PYW07_002895	1281.79	0.00	-12.70	Cuticle protein 8 [*Locusta migratoria]*
CPR48-RR2	PYW07_015450	1187.84	0.00	-12.59	Cuticle protein 7-like [*Spodoptera frugiperda*]
CPR49-RR2	PYW07_015476	7878.56	1.37	-12.48	Cuticle protein 8-like [*Helicoverpa armigera*]
CPR50-RR2	PYW07_015470	970.53	0.00	-12.30	Cuticular protein RR-2 [*Spodoptera litura*
CPR51-RR2	PYW07_015470	970.53	0.00	-12.30	Cuticular protein RR-2 [*Spodoptera litura*
CPR52-RR2	PYW07_008994	8984.16	2.05	-12.09	Cuticle protein 7-like isoform X2 [*Helicoverpa armigera*
CPR53-RR2	PYW07_015461	10158.56	2.40	-12.05	Cuticle protein 8-like [*Helicoverpa armigera*]
CPR54-RR2	PYW07_015446	2447.59	0.69	-11.80	Cuticle protein 8-like [*Spodoptera frugiperda*]
CPR55-RR2	PYW07_002892	609.47	0.00	-11.63	Cuticle protein [*Anopheles gambiae*]
CPR56-RR2	PYW07_015447	3954.65	1.37	-11.49	Larval cuticle protein A1A-like [*Trichoplusia ni*]
CPR57-RR2	PYW07_015465	4050.80	1.41	-11.49	Cuticle protein 8-like [*Spodoptera frugiperda*]
CPR58-RR2	PYW07_015449	2700.37	1.09	-11.29	Cuticle protein 8-like [*Helicoverpa armigera*]
CPR59-RR2	PYW07_015459	442.88	0.00	-11.17	Cuticle protein 19 [*Locusta migratoria*]
CPR60-RR2	PYW07_015474	434.28	0.00	-11.14	Cuticle protein-like [*Spodoptera frugiperda*]
CPR61-RR2	PYW07_015477	8047.07	3.85	-11.03	Cuticle protein 8-like isoform X1 [*Helicoverpa armigera*]
CPR62-RR2	PYW07_015455	3123.22	2.05	-10.57	Cuticle protein 7-like [*Spodoptera frugiperda*]
CPR63-RR2	PYW07_008989	6896.59	4.92	-10.45	Cuticle protein [*Spodoptera exigua*]
CPR64-RR2	PYW07_015464	935.54	0.69	-10.41	Cuticle protein 8-like [*Spodoptera frugiperda*]
CPR65-RR2	PYW07_015451	1341.88	1.03	-10.35	Cuticle protein 7-like [*Spodoptera frugiperda*]
CPR66-RR2	PYW07_015463	1679.07	1.82	-9.86	Cuticle protein 7-like [*Helicoverpa armigera*]
CPR67-RR2	PYW07_015489	167.01	0.00	-9.76	Cuticle protein 19 [*Helicoverpa armigera*]
CPR68-RR2	PYW07_015440	461.60	1.03	-8.79	Cuticle protein 19 [*Trichoplusia ni*]
CPR69-RR2	PYW07_015458	63.00	0.00	-8.35	Cuticle protein 19 [*Helicoverpa armigera*]
CPR70-RR2	PYW07_001952	9898.42	50.70	-7.61	Larval cuticle protein 1 [*Helicoverpa armigera*]
CPR71-RR2	PYW07_001952	9898.42	50.70	-7.61	Larval cuticle protein 1 [*Helicoverpa armigera*]
CPR72-RR2	PYW07_001326	2574.26	15.88	-7.34	Larval/pupal rigid cuticle protein 66 [*Hyalophora cecropia*]
CPR73-RR2	PYW07_015494	23.68	0.00	-6.94	Cuticle protein 19 [*Helicoverpa armigera*]
CPR74-RR2	PYW07_015495	21.67	0.00	-6.81	Cuticle protein 19-like [*Trichoplusia ni*]
CPR75-RR2	PYW07_017317	105.50	1.03	-6.67	Cuticle protein 7-like [*Trichoplusia ni*]
CPR76-RR2	PYW07_017318	100.50	1.03	-6.60	Cuticle protein 7-like [*Trichoplusia ni*]
CPR77-RR2	PYW07_015497	31.77	0.34	-6.40	Cuticle protein 8 [*Helicoverpa armigera*]
CPR78-RR2	PYW07_009001	375.86	6.38	-5.88	Cuticle protein 8 [*Heliothis virescens*]
CPR79-RR2	PYW07_015488	11.21	0.00	-5.87	Cuticle protein 19 [*Helicoverpa armigera*]
CPR80-RR2	PYW07_015141	1271.62	24.62	-5.69	Cuticle protein 8-like isoform X2 [*Spodoptera frugiperda*]
CPR81-RR2	PYW07_015460	9.36	0.00	-5.62	Cuticle protein 8 [*Spodoptera frugiperda*]
CPR82-RR2	PYW07_015492	123.83	2.74	-5.49	Adult-specific cuticular protein ACP-20-like [*Spodoptera litura*]
CPR83-RR2	PYW07_015500	236.53	17.01	-3.80	Cuticle protein 19 [*Heliothis virescens*]

CPR84-RR2	PYW07_016807	101.32	14.70	-2.78	Cuticle protein 8 [*Spodoptera exigua*]
CPR85-RR2	PYW07_016809	100.17	14.68	-2.68	Cuticle protein 8 [*Spodoptera exigua*]
CPR86-RR3	PYW07_001328	152.99	5405.66	5.14	Cuticle protein 16.5-like isoform X1 [*Helicoverpa armigera*]
CPR87-CPH	PYW07_011810	57.28	5050.07	6.46	Pupal cuticle protein PCP52 [*Heliothis virescens*]
CPR88-CPH	PYW07_013820	284.78	15869.90	5.80	Cuticular protein CPH [Spodoptera litura]
CPR89-CPH	PYW07_008540	7856.78	1958.63	-2.00	Pupal cuticle protein [*Heliothis virescens*]
CPR90	PYW07_015486	189.04	0.00	-9.94	Cuticle protein 19 [*Helicoverpa armigera*]

Table S6. List of differentially expressed genes associated with pesticide resistance in the larval integument of solitary and gregarious *M. separata.*

Gene name	gen id	S-RPKM	G-RPKM	Log2 ratio(G/S)	Annotation
P450-1	PYW07_010027	0.00	1571.33	13.14	Cytochrome P450 CYP141 [*Mythimna separata*]
P450-2	PYW07_010028	0.00	1304.08	12.87	Cytochrome P450 CYP141 [*Mythimna separata*]
P450-3	PYW07_007829	1924.12	0.00	-13.29	Probable Cytochrome P450 6a14 [*Spodoptera frugiperda*]
P450-4	PYW07_009868	3286.11	1.46	-11.15	Cytochrome P450 4C1-like [*Spodoptera frugiperda*]
P450-5	PYW07_006327	91.52	0.68	-7.04	Probable Cytochrome P450 49a1 isoform X1 [*Helicoverpa armigera*]
P450-6	PYW07_006329	1327.93	14.51	-6.51	Probable Cytochrome P450 49a1 isoform X1 [*Helicoverpa armigera*]
P450-7	PYW07_002618	134.17	1.71	-6.28	Cytochrome P450 6j1[*Spodoptera exigua*]
P450-8	PYW07_006326	3438.16	90.81	-5.24	Probable cytochrome P450 301a1, mitochondrial [*Spodoptera frugiperda*]
P450-9	PYW07_006328	6.08	0.00	-4.99	Probable cytochrome P450 49a1 [*Spodoptera frugiperda*]
P450-10	PYW07_008367	315.49	27.73	-3.51	Cytochrome P450 4C1 [*Heliothis virescens*]
P450-11	PYW07_015209	452.34	54.13	-3.06	Cytochrome P450 CYP120 [*Mythimna separata*]
P450-12	PYW07_008368	404.34	48.63	-3.06	Cytochrome P450 4C1-like [*Spodoptera litura*]
P450-13	PYW07_007304	1846.67	446.26	-2.05	Cytochrome P450 CYP4A304 [*Mythimna separata*]
P450-14	PYW07_002731	77.81	1552.04	4.31	Cytochrome P450 [*Helicoverpa armigera*]
P450-15	PYW07_016837	51.66	1327.43	4.68	Cytochrome P450 9e2 [*Spodoptera frugiperda*]
P450-16	PYW07_008717	3.97	652.47	7.42	CYP6AE88 [*M. separata*]
P450-17	PYW07_016840	2.92	531.09	7.55	Cytochrome P450 CYP9A100 [*M. separata*]
P450-18	PYW07_005897	584.41	1202.08	1.04	Carbonyl reductase [NADPH] 3-like [*Spodoptera litura*]
P450-19	PYW07_003009	310.52	800.87	1.37	Cytochrome P450 CYP6A100 [*M. separata*]
P450-20	PYW07_002544	1862.51	6282.30	1.75	Cytochrome p450 CYP324A1 [*Spodoptera exigua*]
P450-21	PYW07_002541	71.55	241.30	1.76	Cytochrome P450 CYP6A104 [*Mythimna separata*]
P450-22	PYW07_014206	97.70	338.53	1.79	Cytochrome P450 CYP110 [*Mythimna separata*]
P450-23	PYW07_014208	71.10	257.99	1.85	Cytochrome P450 CYP110 [*Mythimna separata*]
P450-24	PYW07_004528	124.65	484.67	1.96	Cytochrome P450 CYP12A2-like [*Helicoverpa armigera*]
P450-25	PYW07_004574	68.55	293.61	2.11	Cytochrome P450 CYP12A2-like [*Helicoverpa armigera*]
P450-26	PYW07_007301	259.38	1188.32	2.20	Cytochrome P450 monooxygenase CYP4M [*Mamestra brassicae*]
P450-27	PYW07_014186	70.80	372.98	2.39	Cytochrome P450 CYP110 [*M. separata*]
P450-28	PYW07_015822	34.12	194.63	2.50	Cytochrome P450 CYP150 [*M. separata*]
P450-29	PYW07_014183	46.10	265.32	2.52	Cytochrome P450 CYP110 [*M. separata*]
P450-30	PYW07_010038	69.44	406.20	2.55	Cytochrome P450 CYP131 [*M. separata*]

P450-31	PYW07_015820	182.23	1177.16	2.69	Cytochrome P450 CYP150 [*M. separata*]
P450-32	PYW07_016841	148.92	1002.84	2.75	Cytochrome P450 CYP9A100 [*M. separata*]
P450-33	PYW07_002542	138.29	1004.90	2.86	Cytochrome P450 6B2-like isoform X2 [*Spodoptera litura*]
P450-34	PYW07_014316	95.59	720.37	2.91	Probable Cytochrome P450 6a13 [*Helicoverpa armigera*]
P450-35	PYW07_001756	59.04	659.32	3.48	Probable Cytochrome P450 6a13 [*Spodoptera frugiperda*]
P450-36	PYW07_016098	2184.50	28212.11	3.69	Cytochrome P450 4g15 [*Heliothis virescens*]
P450-37	PYW07_013709	84.63	1223.26	3.84	CYP6AB142 [*M. separata*]
P450-38	PYW07_015029	115.13	2937.94	4.67	Cytochrome P450 CYP121 [*M. separata*]
P450-39	PYW07_010039	4.98	173.15	5.09	Cytochrome P450 CYP138, partial [*M. separata*]
P450-40	PYW07_016853	10.57	631.27	5.89	P450 CYP9A134 [*M. separata*]
P450-41	PYW07_002730	15.40	1377.33	6.49	Cytochrome P450 6B6 OS [*Helicoverpa armigera*]
P450-42	PYW07_002641	3.80	352.44	6.58	Cytochrome P450 6k1 [*Blattella germanica*]
P450-43	PYW07_009847	0.73	161.22	8.02	Cytochrome P450 CYP4A314 [*M. separata*]
P450-44	PYW07_010029	0.87	322.53	8.09	P450 CYP131 [*M. separata*]
P450-45	PYW07_010031	0.00	148.82	9.74	Cytochrome P450 CYP131 [*M. separata*]
P450-46	PYW07_009848	0.00	239.54	10.43	Cytochrome P450 CYP4A319 [*M. separata*]
GST-1	PYW07_006140	22.74	2819.24	6.96	Glutathione S-transferase epsilon [*M. separata*]
GST-2	PYW07_015223	72.56	8924.55	6.95	Glutathione S-transferase GST epsilon class [*Spodoptera frugiperda*]
GST-3	PYW07_016690	1019.79	11746.92	3.53	Glutathione S-transferase [*Agrotis ipsilon*]
GST-4	PYW07_006141	3.77	163.35	5.40	Glutathione S-transferase epsilon [*M. separata*]
GST-5	PYW07_005384	6.85	243.20	5.21	Glutathione S-transferase 2-like [*Spodoptera litura*]
GST-6	PYW07_009136	22.22	306.46	3.78	Glutathione S-transferase epsilon 13 [*Spodoptera exigua*]
GST-7	PYW07_005383	0.00	96.14	9.11	Glutathione S-transferase 2-like [*Spodoptera litura*]
GST-8	PYW07_005385	2974.93	33069.22	3.47	Glutathione S-transferase sigma 4 [*Spodoptera exigua*]
GST-9	PYW07_016689	1989.32	106160.74	5.74	Glutathione S-transferase [*Blattella germanica*]
GST-10	PYW07_015222	18.56	189.23	3.33	Glutathione S-transferase GST epsilon class [*Spodoptera frugiperda*]
GST-11	PYW07_003606	775.26	3205.47	2.05	Glutathione S-transferase 1-like isoform X2 [*Spodoptera frugiperda*]
GST-12	PYW07_006139	13.86	217.78	3.98	Glutathione S-transferase epsilon [*M. separata*]
GST-13	PYW07_005380	23.65	188.42	3.01	Glutathione S-transferase sigma 4 [*Spodoptera exigua*]
GST-14	PYW07_006138	0.00	12.86	6.20	Glutathione S-transferase [*Spodoptera litura*]
GST-15	PYW07_016769	1257.34	4711.19	1.91	Glutathione S-transferase delta 3 [*Spodoptera exigua*]
GST-16	PYW07_005634	999.04	202.58	-2.30	Glutathione S-transferase 1, isoform D [*Anopheles gambiae*]
GST-17	PYW07_016464	339.82	841.01	1.31	Glutathione S-transferase 1-1 [*Helicoverpa armigera*]
GST-18	PYW07_016691	151.11	1169.12	2.95	Glutathione S-transferase GSTS2 [*Helicoverpa armigera*]
GST-19	PYW07_014564	461.17	1931.36	2.07	Microsomal Glutathione S-transferase 1-1 [*Spodoptera litura*]
UGT-1	PYW07_010550	75.97	3348.70	5.47	UDP-glucosyltransferase [*M. separata*]
UGT-2	PYW07_001806	56.71	1354.12	4.57	UDP-glucuronosyltransferase 2B31-like [*Spodoptera litura*]
UGT-3	PYW07_009134	263.47	1136.13	2.11	UDP-glycosyltransferase UGT40Q1 [*Helicoverpa armigera]*
UGT-4	PYW07_013032	42.65	253.15	2.56	UDP-glucuronosyltransferase 2B15-like [*Spodoptera frugiperda*]
UGT-5	PYW07_013034	16.86	246.76	3.86	UDP-glucuronosyltransferase 2B15-like [*Spodoptera frugiperda*]
UGT-6	PYW07_001803	5.36	56.39	3.42	UDP-glucuronosyltransferase 2B31-like [*Spodoptera litura*]
UGT-7	PYW07_002077	25.93	117.59	2.20	UDP-glucuronosyltransferase 2B1-like isoform X1 *[Helicoverpa armigera]*
UGT-8	PYW07_007922	73.75	4.79	-3.94	UDP-glucuronosyl and UDP-glucosyl transferase [*Heliothis virescens*]

UGT-9	PYW07_001808	1.08	38.49	4.84	UDP-glucuronosyltransferase 2B31-like [*Spodoptera litura*]
UGT-10	PYW07_004565	3.58	134.77	5.25	UDP-glycosyltransferase 39B4 [*Spodoptera exigua*]
UGT-11	PYW07_001804	0.37	25.04	6.20	Ecdysteroid UDP-glucosyltransferase[*Spodoptera exigua*]
UGT-12	PYW07_004607	3.66	168.40	5.56	UDP-glycosyltransferase 39B4 [*Spodoptera exigua*]
UGT-13	PYW07_009123	4.55	126.21	4.73	UDP-glucuronosyltransferase 2B1-like [*Helicoverpa armigera*]
UGT-14	PYW07_008003	475.05	2577.39	2.44	UDP-glucuronosyltransferase 1-2-like [*Spodoptera frugiperda*]
UGT-15	PYW07_009124	234.10	992.31	2.08	UDP-glycosyltransferase UGT40M1 [*Helicoverpa armigera*]
UGT-16	PYW07_001805	11.21	1566.68	7.12	UDP-glucuronosyltransferase 2B31-like [*Spodoptera litura*]
